# Frequency and correlates of symptoms of anxiety and depression among young caregivers of cancer patients: a pilot study

**DOI:** 10.1186/s13104-018-3740-8

**Published:** 2018-08-31

**Authors:** Muhammad Hassan Majeed, Muhammad Abbas Khokhar, Maryam Abid, Awais Raza, Muhammad Nawaz Qaisar, Ali Ahsan Ali, Ahmed Waqas

**Affiliations:** 1 Department of Psychiatry, Natchaug Hospital, Mansfield, CT USA; 2 Department of Oncology and Radiotherapy, King Edward Medical University, Lahore, Pakistan; 3 King Edward Medical University, Lahore, Pakistan; 4 Faculty of Management Sciences, Hamdard University, Islamabad Campus, Islamabad, Pakistan; 5 Icahn School of Medicine at Mount Sinai, New York, USA; 6 CMH Lahore Medical College & Institute of Dentistry, Lahore Cantt, Pakistan; 7 Human Development Research Foundation, Rawalpindi, Pakistan

**Keywords:** Care-giver, Anxiety, Depression, Cancer, Youth, Young caregivers

## Abstract

**Objectives:**

To determine the frequency of symptoms of anxiety and depression among the young caregivers of family members with cancer and their correlation with role of gender, age and socio-economic status.

**Results:**

A total of 87.8% of caregivers were between 11 and 16 years of age, with 94.6% reported having support from another caregiver. At least 95% of caregivers reported symptoms of anxiety with a higher predisposition among females. Around 73% of caregivers had low monthly incomes followed by (22.9%) middle and (4.1%) high monthly incomes. Care givers belonging to low income groups were more likely to report anxiety and depressive symptoms (70%). Young adults 17–18 years of age reported fewer symptoms of anxiety (10.9%) than their younger counterparts. Reported symptoms of anxiety and depression decreased when the number of care givers increased—2 (67.5%), 3 (16.2%), 4 (5.4%). Increased hospital stay was associated with increased frequency of symptoms, but not beyond 5 weeks.

**Electronic supplementary material:**

The online version of this article (10.1186/s13104-018-3740-8) contains supplementary material, which is available to authorized users.

## Introduction

Life expectancy has increased for people with terminal illnesses, as a result of medical advances and the availability of efficacious palliative treatment. However, this is often associated with psychological and physical burden for family members and caregivers [1, 2]. It is estimated that 4% to 15% of the children in developed nations now live with a seriously sick parent, causing significant psychological distress among their children [3–5]. They worry about their parent’s lives, finances and the future of their family [6].

The expression of these worries may be internalized or externalized, with children feeling scared, angry, guilty and neglected [7]. Internalized symptoms such as anxiety, depression and poor self-esteem and isolation are frequent findings [8, 9]. Externalized symptoms include hyperactivity, impulsivity, decreased interest in sports, academics and aggressive behaviors [10, 11]. All of these symptoms, particularly poor self-esteem, are strongly associated with poor functioning of the children. A child’s reaction to parental cancer is further mediated by their gender and age, for instance, adolescent daughters exhibit more internalized symptoms, particularly anxiety, when compared with adolescent sons [12] and pre-adolescent daughters showed more externalized symptoms, especially poor functioning [13].

Parents usually under-estimate the impact of their disease on their children. Recent literature suggests a problem-solving approach and open communication, are healthy coping strategies and have yielded better outcomes [14–16]. Whereas avoidance, wishful thinking, isolation and denial are associated with poor psychological outcomes among children [14–16]. Adolescents and young adults who have a more open communication with their parents about their pathology exhibit better functioning [17, 18].

Parentification—role reversal where a child is obliged to act as a parent to their own parent—is another strategy for families dealing with parental cancer [15, 19]. In this approach, children take care of their parents’ physical requirements often at the cost of their own developmental and psychological needs. This can be physically and psychologically taxing on both the children and parents and may turn out to be counter-productive. Parents’ poor coping with their cancers is also a predictor of poor functioning among their children. On the other hand, parental cancer may lead to some positive attributes in family functioning, including tightening of family bonds and better appreciation of the value of life [19, 20]. These children may develop positive attributes such as autonomy, kindness, empathy and an accelerated psychological development, self-reliance and resilience [21, 22].

Distress among young caregivers of parents with cancers is even more debilitating in low and middle-income countries (LMIC) due to several socio-economic factors. There is a wide gap in the medical literature about the number of these caregivers and their psycho-social issues in a LMIC like Pakistan. In an attempt to fill this gap, we conducted a survey of adolescents and young adults who were caregivers for their terminally ill cancer parents. The purpose of this study is to determine the frequency of psychiatric illness and its correlates such as gender, age, income levels and duration of stay at the hospital among the young caregivers of family members with cancers. Moreover, this pilot study has also been designed to provide sample size and power estimates for future studies.

## Main text

### Methods

A cross-sectional study was conducted over a period of 8 months from January to July 2017 at Mayo Hospital, Lahore, Pakistan. Mayo Hospital serves a population of over ten million in a major metropolitan center.

A non-probability sampling method was used to select the children of 90 patients receiving treatment for cancers from both inpatient and outpatient clinical settings of the Department of Oncology at the Mayo Hospital. Ethical approval was sought from and granted by ethical review committee of the King Edward Medical University, Lahore, Pakistan. The selection included respondents from both genders, ranging in age from 11 to 21 years. Enrollment in this study was voluntary and based on written informed consent. For adolescents and young adults below 18 years of age, informed assent was also taken from their parents.

Participants were interviewed using a questionnaire consisting of three parts: (a) demographics (b) Urdu translations of the self-report mood and feeling questionnaire (MFQ) short version, and (c) the Spence Children Anxiety Scale (SCAS) [23, 24].

The respondents were assessed for symptoms of depression using the 13 item MFQ (Additional file 1: Dataset S1) that rates depressive symptoms on a three point Likert scale as 0 (not true) to 2 (true) [23]. This scale has shown adequate validity and reliability among the Urdu speaking population in Pakistan. Moreover, Kent et al. [23] have reported an acceptable sensitivity (67.1%), specificity (80%), positive predictive value (77%) and negative predictive value (71%) in screening depression among children using this scale.

Symptoms of anxiety were assessed using the SCAS among children and adolescents (Additional file 2: Dataset 2). It comprises of a total of 38 questions with responses recorded on a 4-point Likert scale ranging from 0 (never) to 3 (always) [24]. It has shown an excellent dimensionality and internal consistency with a co-efficient alpha of 0.92 and a Guttman split half reliability of 0.90 [24].

Apriori sample size calculation was calculated using GPower (v3.1.7). Based on number of predictors (5), desired power 80%, an expected moderate effect size (f_2_ = 0.15) and a significance level of 0.05, a minimum sample size of 90 was required. Out of the initial sample of 90 patients, 15 respondents were excluded because of incomplete or incorrect information and the remaining (75) were included in the final analysis. All data were analyzed using SPSS v.20. Descriptive statistics were recorded for quantitative and categorical variables. Chi square was used to assess association between two categorical variables. Pearson correlation was used to assess association between two quantitative variables.

### Results

The study sample comprised of male (41.9%) and female (58.1%) caregiving children living with their mothers (39%), fathers (32%), siblings (1.4%), and grandparents (2.7%) who were under inpatient and outpatient cancer treatment. The vast majority, 87.8%, of caregivers were of age 11–16 years, indicated their household income levels as low (73%), middle (22.9%), and high (4.1%). Most caregivers had other relatives to support their caregiving task (2 caregivers = 73% and 3 caregivers = 16.2%). A total of 63.5% of caregivers indicated a total inpatient stay of up to 4 weeks (Table 1).

**Table 1:** Descriptive statistics

Variable description	Frequency (n)	Percent (%)
Gender		
Male	31	41.9
Female	43	58.1
Age group (years)		
11–13	32	43.2
14–16	33	44.6
17–18	9	12.2
Patient’s relationship with caregiving child		
Mother	39	52.7
Father	32	43.2
Sibling	1	1.4
Grandparent	2	2.7
Number of caregivers		
1 person	4	5.4
2 persons	54	73.0
3 persons	12	16.2
4 persons	4	5.4
Income group		
Low (< Rs. 20,000)	54	73.0
Middle (Rs. 20,001–40,000)	17	22.9
High (Rs. 40,001–60,000)	3	4.1
Duration of total stay in hospital (weeks)		
≤ 2	21	28.4
3–4	26	35.1
≥ 5	27	36.5

About 95% of the young caregivers had symptoms of anxiety while 9.4% showed depression symptoms (Table 2). Female caregivers had a higher frequency of anxiety (54%) and depression (6.7%) than the male caregivers (p > 0.05).

**Table 2:** Correlates of symptoms of anxiety and depression among pediatric caregivers

	Anxiety^a^		Chi square	Depression^b^		Chi square	Total
	No	Yes		No	Yes		
Gender							
Male	1	30 (41%)	χ^2^ (1, N = 74) = 0.496, p = 0.481	29	2 (2.7%)	χ^2^ (1, N = 74) = 0.564, p = 0.453	31 (41.9%)
Female	3	40 (54%)		38	5 (6.7%)		43 (58.1%)
Total	4 (5%)	70 (95%)		67 (90.6%)	7 (9.4%)		74 (100%)
Age groups (years)							
11–13	1	31 (41.9%)	χ^2^ (2, N = 74) = 0.926, p = 0.629	29	3 (4.0%)	χ^2^ (2, N = 74) = 2.157, p = 0.340	32 (43.2%)
14–16	2	31 (41.9%)		31	2 (2.7%)		33 (44.6%)
17–18	1	8 (10.9%)		7	2 (2.7%)		9 (12.2)
Total	4 (5%)	70 (95%)		67 (90.6%)	7 (9.4%)		
Number of caregivers							
1 person	–	4 (5.4%)	χ^2^ (3, N = 74) = 1.566, p = 0.667	4	–	χ^2^ (3, N = 74) = 2.972, p = 0.396	4 (5.4%)
2 persons	4	50 (67.5%)		48	6 (8.1%)		54 (72.9%)
3 persons	–	12 (16.2%)		12	–		12 (16.2%)
4 persons	–	4 (5.4%)		3	1 (1.3%)		4 (5.4%)
Total	4 (5%)	70 (95%)		67 (90.6%)	7 (9.4%)		74 (100%)
Income levels							
Low (< Rs. 20,000)	2	52 (70.2%)	χ^2^ (2, N = 74) = 1.822, p = 0.402	49	5 (6.8%)	χ^2^ (2, N = 74) = 2.253, p = 0.324	54 (73.0%)
Middle (Rs. 20,001–40,000)	2	15 (20.3%)		16	1 (1.3%)		17 (23.0%)
High (Rs. 40,000–60,000)	0	3 (4.5%)		2	1 (1.3%)		3 (4.0%)
Total	4 (5%)	70 (95%)		67 (90.6%)	7 (9.4%)		74 (100%)
Low (< Rs. 20,000)	2	52 (70.2%)		49	5 (6.8%)		54 (73.0%)
Stay in hospital (weeks)							
< 2	0	21 (28.4%)	χ^2^ (2, N = 74) = 7.361, p < 0.025	20	1 (1.3%)	χ^2^ (2, N = 74) = 4.484, p < 0.100	21 (28.4%)
3–4	0	26 (35.1%)		21	5 (6.8%)		26 (35.1%)
> 5	4	23 (31.0%)		26	1 (1.3%)		27 (36.5%)
Total	4 (5%)	70 (95%)		67 (90.6%)	7 (9.4%)		74 (100%)

^a^Anxiety (SCAS score > 12 = yes, < 12 = no)

^b^Depression (MFQ score; male: > 33 = yes, < 33 = no; female: > 39 = yes, < 39 = no)

It was observed that 84% of young care givers of 11–16 years of age had anxiety symptoms while only 7% had depressive symptoms (Table 2). Those between 17–18 years of age reported lesser symptoms of anxiety, however, this was not significant (p > 0.05).

Anxiety and depressive symptoms were reported less frequently among caregivers as the number of caregivers for a patient increased—2 caregivers 67.5%; 3 caregivers 16.2%; 4 caregivers 5.4% (Table 2). Depressive symptoms were less reported, 8.1% when 2 care givers were involved (p > 0.5). However, these results did not exhibit statistical significance.

It was observed that 70% of young caregivers who reported anxiety symptoms belonged to a lower monthly income group (≤ Rs. 20,000), 20.3% from middle income group (Rs. 20,001–40,000), and 4.5% from higher income group (40,001 to Rs. 60,000) (Table 2).

Duration of hospital stay and its relationship with anxiety and depression was also explored (Table 2). Around 28% of caregivers with ≤ 2 weeks of inpatient hospital stay reported anxiety symptoms, 35.1% with 3–4 weeks of stay and 31% of those who stayed ≥ 5 weeks. And 8.1% of caregivers who stayed in hospital for 3 or more weeks reported depression symptoms (p < 0.1).

A descriptive analysis indicated that the majority of young caregivers reported social phobia (67, 90.5%), obsessive compulsive symptoms (61, 82.4%) and generalized anxiety (63, 85.1%), separation anxiety (8.1%), agoraphobia (5, 6.8%).

A significant univariate association was found between symptoms of anxiety among care givers and overall duration of stay at a hospital. However, this association was rendered insignificant when adjusted for age, gender and income levels in a regression model (R^2^ = 0.33, F = 0.60, df = 73, p = 0.67). Table 3 presents these results in detail.

**Table 3:** Predictors of symptoms of anxiety among the young caregivers

Variables	Unstandardized coefficients		Standardized coefficients	*t*	*p*
	B	Std. error	Beta		
(Constant)	53.288	12.305		4.331	< 0.001
Age	− 0.583	0.722	− 0.097	− 0.807	0.422
Gender	0.960	2.815	0.041	0.341	0.734
Income	− 0.355	3.150	− 0.014	− 0.113	0.911
Duration of stay	− 0.175	0.145	− 0.144	− 1.206	0.232

### Discussion

Our study indicates that a high percentage of adolescents and young adults who provide care to relatives with cancer report anxiety spectrum disorders. Symptoms of social phobia and generalized anxiety disorders were the most common psycho-pathologies identified in these subjects. Moreover, a high percentage of participants were screened as having significant depressive symptoms. Adolescent females, from a lower socio-economic status, with poor social support and a relatively longer hospital stay reported a higher frequency of psychiatric morbidity.

Female caregivers presented with more symptoms of anxiety and depression than their male counterparts. These findings are in accordance with previous studies that demonstrate that female adolescent care-providers were more prone to internalizing problems, particularly symptoms of anxiety [12]. Other studies have suggested that pre-adolescent daughters expressed more externalizing problems and poor social functioning. [13].

As expected, the younger respondents exhibited a higher frequency of anxiety symptomatology than older respondents, albeit statistically insignificant. Our results are in accordance with previous literature reporting an age dependent improvement in positive coping skills among children with sick family members [13]. Pre-adolescent and latency-phase children are prone to more psychological and behavioral problems as compared to adolescents [12]. There could be several reasons for this. Parents are more likely to have an open and age-appropriate discussion about their cancers with older children that help with family functioning [15]. Moreover, older children might develop a stronger support network, resilience and self-reliance [22].

The present analysis reports a lower frequency of psychiatric symptomatology among participants with a higher socioeconomic status. In general, children from financially comfortable families have been found to be more resilient than their counterparts [25]. Such families have better resources to deal with the illness and can afford better treatment as well. Similarly, adolescents from families with multiple care providers for the sick member show more resilience, better functioning and lower scores on anxiety scales. Our study is in accordance with the findings of socio-demographic and resource on the caregivers of the cancer patients [26, 27].

As compared to symptoms of anxiety, only 9.4% of the sample showed depressive symptoms. This is comparable to the community sample of a national co-morbidity survey of adolescents in which 11% of the population showed life-time depression [28]. There could be a cultural explanation of this. In Pakistan, people often think of depression as a sign of weakness, even a sin and maybe reluctant to disclose this information.

There are practical implications of these findings. Firstly, high-risk groups should be identified and screened for psychiatric disorders. Secondly, culturally sensitive psycho-social interventions should be designed to prevent and treat psychiatric morbidities among young caregivers. In the absence of specialized programs, clinicians can aid children meet their psychosocial needs. Providing a safe and open space for expressing their emotions and starting an age-appropriate communication on cancer, treatment and prognosis of the patient can help these children to cope better with distress [29]. By participating in group and individual therapy these caregivers can also learn psychological skills to deal with their own psychological distress. Moreover, treating oncologists may also need to consider that longer stays in the hospital may be precipitating and perpetuating, anxiety disorders in young caregivers.

### Conclusion

There is a high frequency of symptoms of anxiety and depression among young care givers of cancer patients. Due to the laborious nature of care often required by patients suffering from cancer, parents and family members may the psychological needs of these adolescents.

## Limitations

We acknowledge a few limitations of this study. First, other than length of hospital stay, variable results for age, gender, and social support and income levels did not reach significance. This could likely be due to a small sample size leading to a poor statistical power. However, this study provides a useful resource to base future power calculations on.

Our sample was also noted for minimal ethnic diversity, with convenience sampling style, cross-sectional design of the study, self-reporting nature of the questionnaires and a single location for recruitment of the subjects, limiting its generalizability. Second, our analysis did not account for the type, prognosis and stage of the diagnosed cancer. Moreover, our study did not explore the relationship of the time since the diagnosis of cancer and the course of psychological distress in caregivers as it may fluctuate during course of treatment. Well-designed and multi-center studies taking into account above limitations are encouraged in future.

## Additional files

**Additional file 1: Dataset S1.** This data file contains variables pertaining to depressive symptoms of respondents.

**Additional file 2: Dataset S2.** This data file contains variables pertaining to anxiety symptoms of respondents.

## Abbreviations

LAMI: low and middle income
MFQ: mood and feeling questionnaire
SCAS: the Spencer Children Anxiety Scale
